# A Review of the Literature Regarding Sleep and Cardiometabolic Disease in African Descent Populations

**DOI:** 10.3389/fendo.2018.00140

**Published:** 2018-04-11

**Authors:** Peter L. Whitesell, Jennifer Obi, Nuri S. Tamanna, Anne E. Sumner

**Affiliations:** ^1^Howard University Hospital Sleep Disorders Center, Washington, DC, United States; ^2^Department of Internal Medicine, Howard University Hospital, Washington, DC, United States; ^3^Section on Ethnicity and Health, Diabetes, Endocrinology and Obesity Branch, National Institute of Diabetes, Digestive and Kidney Diseases and National Institute of Minority Health and Health Disparities, National Institutes of Health (NIH), Bethesda, MD, United States

**Keywords:** sleep disturbances, insomnia, obstructive sleep apnea, obesity, African-Americans, Africans

## Abstract

In the twenty-first century, African descent populations on both the continent of Africa and throughout the world are experiencing a high rate of both sleep disturbances and cardiometabolic diseases. The most common sleep disturbances are reduced sleep duration, insomnia, disordered circadian rhythm, and obstructive sleep apnea. Cardiometabolic diseases include hypertension, coronary artery disease, diabetes, hyperlipidemia, and the metabolic syndrome. This review seeks to call attention to new insights regarding the impact of sleep disturbance on cardiometabolic risk factors and outcomes and then apply these concepts to African descent populations, a relatively understudied population. Initial data suggest disparities in sleep quality may have an important role in current and emerging patterns of cardiometabolic disease for African descent populations both in the United States and abroad.

**ClinicalTrials.gov Identifier:**

Not applicable.

## Introduction

African descent populations have a high prevalence of both sleep disturbances and cardiometabolic disease (1–6). Sleep disturbances include reduced sleep duration, insomnia, disordered circadian rhythm, and obstructive sleep apnea (OSA). The term cardiometabolic disease encompasses hypertension, coronary artery disease, diabetes, and hyperlipidemia, all of which are associated with adverse cardiovascular outcomes and the metabolic syndrome. In this review, we present evidence which suggests that sleep disturbances may be a key factor contributing to the development of cardiometabolic disease in populations of African descent.

This review is divided into four parts. First, the tools used to evaluate sleep are described. Second, the physiologic and epidemiologic evidence correlating impairment of sleep to adverse cardiometabolic disease effects is presented. In specific, the data regarding both reduced habitual sleep duration and disorders of sleep are reviewed (Tables 1 and 2). Third, the data relevant to African-Americans (AAs) are described. Overall, sleep impairment may have an important role in the disparities in cardiovascular outcomes experienced by the AA population. Finally, we review the sparse literature on sleep in Africans. In view of the convincing connection between sleep and cardiometabolic disease, evolving adverse trends in sleep in Africa may have important implications for the cardiovascular health of this understudied population, the second most populous continent in the world (7).

**Table 1:** Summary of studies examining sleep duration and cardiometabolic diseases in African-Americans (AAs).

Reference	Type of study	Subjects/number of studies	Results
Knutson et al. (72)	Cohort study and longitudinal, blood pressure and actigraphic sleep measured at 5-year intervals	578 AA and White	Reduced sleep predicted higher BP and adverse change in BP; sleep duration appeared to mediate racial differences in diastolic BP

Knutson (54)	National surveys, self-report	8 surveys conducted over 31 years	AAs consistently more likely to report short sleep duration than any other racial group

Vishnu et al. (73)	Cross-sectional survey, self-report	369,217 multi-ethnic US subjects	Strongest correlation between insufficient sleep and CHD found in AAs

Ruiters et al. (66)	Meta-analysis, variable methods including subjective and objective measures of sleep	14 studies	AAs had poorer sleep continuity and duration, less slow wave sleep, and a greater proportion of stage 2 sleep

Jean-Louis et al. (74)	National Health Interview Survey, 1977–2009, cross-sectional, face to face interviews	All ethnicities surveyed but restricted to Black and White participants	Among Black, very short sleepers 76% increased risk of overweight and 81% increased risk of obese

Halder et al. (68)	Cohort study using PSQI and in home polysomnography, correlation with % African genetic ancestry	70 AA and 101 European-American	% African ancestry correlated with lower percentage slow wave sleep but not sleep duration or efficiency

Curtis et al. (63)	Cross-sectional, actigraphic sleep	426 Midwestern US adults with oversampling of AAs	AA obtained less sleep and had lower sleep efficiency. Sleep duration predicted 41% of cardiometabolic risk factor variance

Johnson et al. (67)	Cross-sectional analysis, self-report	5,301 AAs in the Jackson Heart Study	54% reported <6 h sleep, 5% >9 h sleep. Social and environmental characteristics associated with sleep duration

Riestra et al. (70)	Cross-sectional analysis	2,962 AA participants	A SNP in the CLOCK gene associated with sleep duration after multifactorial adjustments

**Table 2:** Summary of studies examining association between sleep disorders and cardiometabolic diseases in African-Americans (AAs).

Reference	Type of study	Follow-up period/no of studies	Results
Kripke et al. (79)	Prevalence survey of sleep-disordered breathing (SDB) in San Diego adults	1 year	Survey indicated SDB is more common, among minorities, approximately threefold in Blacks versus Whites

Jean-Louis et al. (76)	Stratified cluster sampling community dwelling AA and European-American older adults, 1,118 subjects	Single survey	Sleep disturbance varied both between ethnicities and within ethnicities

Ruiter et al. (75)	Meta-analysis of differences between AAs and Caucasian-Americans in insomnia symptoms and SDB	13 studies measuring insomnia and 10 measuring SDB	Significant increase in prevalence and severity in AAs versus whites after adjustment for age, gender, and body mass index

Lieu et al. (86)	Prospective study examining the association between rotating night shift work and incident hypertension in 1,510 Black and 94,?142 White females in the Nurses′ Health Study II	16 years	Rotating night shift work was independently associated with an increased risk of hypertension in Blacks but not in Whites

Kingsbury et al. (2)	Review of sleep and cardiovascular outcomes (e.g., hypertension, stroke, cardiovascular disease) in relation to racial/ethnic differences	Multiple	Discussed potential mechanisms for sleep disorders, such as SDB and insomnia, to influence the pathogenesis of CVD

Sands et al. (87)	Self-report data from National Health and Nutrition Examination Survey, 2007–2008	Single survey	Probable sleep apnea more strongly associated with hypertension in overweight AAs (4.7-fold risk) versus overweight Whites (1.6-fold risk) or obese Hispanic/Latinos (2.0-fold risk)

Williams et al. (81)	Systematic review of potential ramifications of inadequate sleep in a multicultural context	Multiple studies	Severity of sleepiness corresponded to levels of the proinflammatory cytokine IL-6. Evidence that the majority of AAs referred for evaluation of sleep apnea do not complete testing or are not compliant with treatment

Jean-Louis et al. (82)	Two-arm randomized, controlled trial of tailored telephone messaging to improve adherence in 380 blacks with metabolic syndrome and risks for sleep apnea	Single intervention	Adherence to consultation appointment improved 3.2-fold with intervention; high rate of compliance with continuous positive airway pressure treatment for both groups once diagnosed

Prasad et al. (80)	Prospective study of 283 patients with newly diagnosed obstructive sleep apnea (OSA) by polysomnography (AHI ≥5 per hour)	2 weeks	AA race, short sleep duration, chronotype, and increased proinflammatory cytokine IL-6 level were associated with sleepiness in OSA

## Part 1: Tools for Sleep Assessment

A variety of instruments are utilized to assess sleep. Validated questionnaires and surveys of self-reported sleep parameters such as typical quantity, latency to sleep onset, perceived degree of sleep disruption and quality, and level of daytime sleepiness are available, such as the Pittsburgh Sleep Quality Index (PSQI) and the Epworth Sleepiness Scale (8, 9). While questionnaires are relatively easy and inexpensive to administer, methodological concerns arise including variations in cultural interpretation by different populations (10). Importantly, there may be major differences in outcomes when comparing subjective versus objectively recorded sleep (11). For example, Bathgate et al. reported that individuals with insomnia and short sleep duration of <6 h as identified by sleep laboratory determined data were found to have a 3.5-fold higher risk of hypertension compared to individuals with insomnia with in laboratory sleep duration ≥6 h (11). However, no significant risk was observed using subjectively determined total sleep time (11).

Polysomnography conducted in a sleep laboratory setting provides detailed nocturnal physiologic data including objective sleep architecture recording and cardiopulmonary monitoring. While polysomnography provides more complete physiologic data than many other techniques, polysomnography has the drawback of expense and assessment of sleep in an atypical environment. For example, changes in laboratory recorded sleep architecture are known to occur on repeated nights of testing and are generally referred to as the “first night effect” (12). Home sleep testing is an alternative with a more natural setting and less resource utilization but may suffer from lower accuracy and less control over data quality (13). Actigraphy evaluates sleep versus wake time with a small wrist worn monitor and can more conveniently capture information over periods as long as several weeks but has been found to only partially correlate with sleep determined by polysomnography (14).

A variety of strategies may be applied in utilizing these tools in the assessment of African descent populations. Agyemang et al. note that a majority of studies evaluating public health problems in migrants rely only on a comparison with the outcomes for the population with the host country of relocation. They argue that additional valuable information can be obtained when analysis is extended across national boundaries to also compare migrants’ health outcomes with compatriots who did not migrate and migrants who are resident in different host countries (15).

## Part 2: Physiologic and Epidemiologic Connections Between Sleep and Cardiometabolic Disease

Sleep is restorative and has a major influence on protein synthesis, hormone release, and autonomic activity (16). Decreased sleep duration or sleep fragmentation adversely affects physiologic processes occurring in the hypothalamic–pituitary–adrenal axis (HPA), as in glucose and lipid metabolism, cardiac function, and vascular control (17). Deficient sleep, either in quantity or quality, has been correlated with a number of adverse cardiometabolic outcomes in both the laboratory and population-based studies (18). In fact, several systematic reviews of epidemiologic data have been published linking sleep disorders and cardiometabolic disease (19, 20). Results of some of these important studies are provided below.

### Sleep Duration and Hypertension

Experimental sleep restriction in adults promotes increases in blood pressure, including nocturnal hypertension. Normally blood pressure decreases by approximately 10% during the night, a pattern referred to as “dipping” (21). In healthy young volunteers, dipping is attenuated under conditions of sleep restriction (21). With chronic sleep restriction, there is enhanced release of plasma and urinary norepinephrine and decreased heart rate variability suggesting activation of the sympathetic nervous system (SNS) (19, 22, 23).

A very large population study, specifically the National Health Interview Survey (NHIS), assessed self-reported sleep duration and hypertension in 71,455 individuals and found a progressive increase in the prevalence of hypertension as sleep duration declined below 7 h (24). The survey identified a fully adjusted hazard ratio of 1.32 for incident hypertension in the group who were sleeping 5 h or less compared to the group which was sleeping 7–8 h (25).

Data also indicate associations between habitual long sleep duration and prevalent hypertension. The NHIS cited above found a hazard ratio for hypertension of 1.30 for individuals sleeping more than 9 h nightly (21). However, the association between long sleep and new incident hypertension is less clear as meta-analysis reviews have failed to confirm a relationship (20).

### Sleep Disorders and Hypertension

Disorders of sleep quality, such as OSA, also promote hypertension. There are a number of mechanisms by which OSA is linked to hypertension. Recurring arousals and sleep fragmentation lead to increased SNS activation, increased levels of catecholamines, and increased cortisol release (19). In addition, there is evidence in OSA for activation of the renin–angiotensin–aldosterone system (26). Furthermore, intermittent hypoxemia for a 6-h interval in healthy volunteers has been shown to increase blood pressure, presumably from elevated sympathetic activation and norepinephrine levels, and this can effect can be prevented by losartan, an angiotensin receptor blocker (27).

Approximately 50% of individuals with OSA are hypertensive and the association between sleep apnea and both systolic and diastolic blood pressure persists even after adjustment for key confounders including obesity (28). A non-dipping blood pressure pattern is correlated with OSA severity indices (i.e., apnea–hypopnea index) and severity of intermittent hypoxemia (29, 30). An important meta-analysis has indicated that treatment of sleep apnea leads to reductions in both diastolic and systolic blood pressure (19).

Insomnia, restless leg syndrome, and periodic limb movement are also correlated with hypertension, although a causal relationship has not been established (31, 32).

It appears likely that many other forms of sleep disturbance besides OSA have the potential to promote hypertension. Sherwood et al. found that non-dipping was correlated with subjective sleep quality as measured by the PSQI and sleep quality as measured by actigraphy, independent of body mass index (BMI) (33). Circadian misalignment, such as occurs in shift workers, is also associated with adverse effects on blood pressure (34).

### Sleep Duration and Cardiovascular Disease

A reduced quantity of sleep results in shorter periods of the lowered blood pressure, heart rate, and sympathetic tone that accompany normal sleep. Furthermore, both endothelial dysfunction and impaired vasodilation occur during conditions of sleep restriction (35). For example, a single 24-h work-shift period in medical residents has been demonstrated to reduce flow-mediated dilation, an indicator of endothelial function (36). One night of sleep deprivation in healthy volunteers resulted in increased arterial stiffness as reflected by changes pulse wave velocity (37), impaired left ventricular diastolic function, and increased QT intervals (38). In addition, sleep restriction is associated with elevated levels of inflammatory cytokines implicated in atherogenesis such as C-reactive protein (CRP), tumor necrosis factor (TNF), and interleukin 6 (IL-6) (31).

Prospective epidemiologic studies have demonstrated that habitual sleep duration correlates with both coronary heart disease and coronary heart disease mortality (20). A meta-analysis found that short sleep duration was associated with a risk ratio of 1.48 (95% CI 1.22–1.8) for cardiovascular events (39) and a prospective Dutch study reported that sleeping more than 7 h lowered the risk of cardiovascular events by 65% and the risk of fatal cardiovascular events by 83% (40).

Similar to hypertension, a U-shaped curve has been found regarding the prevalence of cardiovascular disease and sleep duration with long duration sleep also associating with increased risk. In the NHIS study, those sleeping more than 9 h exhibited an increased risk for MI and, in a 10-year longitudinal study of over 70,000 nurses, those sleeping nine or more hours had an increased 1.4-fold risk of cardiovascular disease (21). In meta-analysis, long sleep duration carried a 1.65 times higher relative risk of incident stroke (20). A recent systematic review of the association between sleep duration and non-invasive markers of subclinical cardiovascular disease found long sleep duration associating with coronary artery calcium and carotid intima-media thickness (41).

### Sleep Disorders and Cardiovascular Disease

Short sleep duration is related to but distinct from insomnia, a disorder of perceived difficulty in sleep initiation, consolidation, duration, or quality. Chronic insomnia is viewed as a state of hyperarousal and abnormalities in both the HPA axis and the SNS have been identified. In the evaluation of sleep disorders, a number of large prospective population studies have been conducted, such as the Taiwan National Health Insurance Research Database which followed over 40,000 people for 10 years (42). A diagnosis of insomnia was associated with a 68% increased incidence of myocardial infarction and an 85% increase in risk of stroke (43).

Obstructive sleep apnea is characterized by increased inspiratory efforts with an occluded or partially occluded airway resulting in cyclical negative intrathoracic pressures and intermittent hypoxia as well as fragmentation of sleep architecture. This adversely effects myocardial contractility and endothelial function and leads to oxidative stress and increased activity of inflammatory pathways (19). As a consequence, OSA is an independent risk factor for incident coronary artery disease, heart failure, and stroke (4). For example, the Sleep Heart Health Study found OSA was associated with increased coronary artery disease, stroke, and all-cause mortality (21).

Other sleep disorders are associated with cardiovascular disease. In a review of non-invasive markers of subclinical cardiovascular disease, poor subjective sleep quality as reflected in the PSQI correlated with measures of endothelial dysfunction and arterial stiffness (41). Circadian misalignment also appears to influence the metabolic and hormonal mechanisms which promote cardiometabolic disease (21). Therefore, it is not surprising that an increased incidence of coronary artery disease has been detected in shift workers (44).

### Sleep Duration and Diabetes, Obesity, and the Metabolic Syndrome

#### Association With Diabetes

A number of physiologic changes, which promote hyperglycemia, correlate with experimental sleep restriction. These include higher evening cortisol levels, increased SNS activity, and catecholamine production, as well as higher levels of inflammatory factors, which promote insulin resistance such as interleukin (IL)-1β, IL-6, IL-17, and CRP (18). Studies have reported alterations in glucose regulation during partial sleep restriction in experimental conditions. For example, Spiegel et al. evaluated glucose tolerance in 11 healthy young men after exposure to 5 days of 4-h sleep durations and found a 40% reduction in glucose tolerance to intravenous glucose and a 30% reduction in the acute insulin response to glucose (45).

This has led many investigators to propose that chronic insufficient sleep, which seems endemic in modern society, may play a major role in the increasing prevalence of diabetes (46). Both prevalent and incident diabetes have been found to correlate with short sleep in epidemiologic surveys (47). In addition, two meta-analyses found that there is approximately a 30% increased risk of diabetes in short sleepers after adjusting for confounders, although in one this effect was only evident in men (48, 49).

Long habitual sleep duration also seems to correlate with increased risk for diabetes. Similar to the pattern seen with hypertension and cardiovascular disease, in the NHIS, sleep duration was found to have a U-shaped relationship to self-reported diabetes (50). One meta-analysis of six studies found a 1.48 increase in the relative risk for new development of diabetes in long sleepers (20). These studies have not adjusted for the presence of snoring and sleep apnea, however, a likely confounding variable.

#### Association With Obesity

Considerable research has been directed toward investigating the link between reduced habitual sleep duration and obesity. For example, in a controlled setting with unrestricted food access, healthy subjects restricted to less than 5 h over a 2-week interval demonstrated a slight increase in calorie metabolism, approximately 111 cal daily (51). Dietary caloric intake, however, greatly exceeded this increased utilization with sleep restriction promoting an average of 2 lbs increased weight weekly (51). Functional brain imaging under conditions of sleep restriction demonstrates reduced activity in cortical areas of the frontal lobe which participate in decision-making and enhanced activity in regions such as the amygdala, which is involved in emotional regulation (52). This is thought to drive increased consumption of caloric rich foods in a sleep-deprived condition (51).

An extensive body of epidemiologic data connects habitual short sleep duration to obesity (53, 54). The Wisconsin Sleep Cohort Study found both short and long sleep durations are linked to an increased risk of a higher BMI; the lowest BMI was seen in individuals sleeping for an average of 7.7 h per night. Additionally, short sleepers had reduced leptin levels, a hormone which promotes satiety, and elevated ghrelin levels, a hormone which promotes appetite (53). A 2008 meta-analysis of epidemiological studies that encompassed 634,511 participants (30,002 children and 604,509 adults) aged 2 years to 102 years, found short sleep duration was associated with an odds ratio of 1.55 (1.43–1.68) in adults and 1.89 (1.46–2.43) in children (48). In a 6-year longitudinal follow-up study, short sleepers (≤6 h) were more likely to accumulate visceral adipose tissue (assessed by computerized tomography) than those sleeping 7 to 8 h (54). Furthermore, a negative linear association was found between sleep duration and both BMI and waist circumference (54).

#### Association With the Metabolic Syndrome

Hypertension, visceral adiposity, hyperglycemia, and dyslipidemia in combination contribute to form the Metabolic Syndrome. The association of short sleep to these first three components has been described above. The influence of reduced sleep on lipids is less certain as the results across studies have been inconsistent (19). However, given evidence linking the hypertension, visceral adiposity, and hyperglycemia, an epidemiologic connection linking short sleep to the Metabolic Syndrome is considered to be strong (54). In the Adult Health and Behavior Project registry, a community-based cross-sectional study of over 1,200 adults, being a short sleeper increased the odds of having the Metabolic Syndrome by 45% (55). A meta-analysis of 18 studies encompassing 75,657 participants found an inverse dose–response relationship between sleep duration and the presence of the Metabolic Syndrome (56).

### Sleep Disorders and Diabetes, Obesity, and the Metabolic Syndrome

#### Association With Diabetes

A large body of literature has demonstrated OSA as a factor in insulin resistance and diabetes, independent of the common cofactor of obesity (19). Intermittent hypoxia and sleep fragmentation can lead to decreases in insulin sensitivity with impairment of beta cell function (57). Potential underlying mechanisms are similar to those for short sleep duration with the added factor of oxidative stress promoting inflammation through the elevation of inflammatory mediators, such as CRP and proinflammatory cytokines, increased corticosteroid levels, sympathetic activation, and catecholamine elevations (19). Cross-sectional studies have demonstrated that patients with OSA have an increased risk of diabetes independent of obesity (relative risk 2.3 in one study) (58) and that patients with diabetes have an increased risk of OSA (e.g., a prevalence between 58 and 86%) (59). A recent population study found a 30% higher incidence of diabetes in those with severe OSA when followed over a 5-year interval (60).

Epidemiological studies of circadian disruption have been conducted in shift workers demonstrating evidence of impaired glucose tolerance and a higher incidence of diabetes in shift workers compared to daytime workers (19). While in part this may reflect reduced sleep duration and/or degradation of sleep architecture with reduced deep sleep (i.e., slow wave, N3 sleep), some evidence indicates that dyssynchrony between the central suprachiasmatic nucleus and peripheral circadian oscillators may have direct effects on metabolism to promote altered glucose metabolism (34).

#### Association With Obesity

Approximately 70–80% of patients with OSA are obese and it is clear that obesity plays a pathogenic role in the development of the condition, as sleep apnea can improve with weight reduction. The degree to which OSA may predispose to weight gain is less certain and most data show a small increase in weight occurs after effective treatment with OSA with continuous positive airway pressure treatment (CPAP) (61).

#### Association With the Metabolic Syndrome

Intermittent hypoxia associated with OSA is thought to lead to upregulation of hepatic synthesis of lipids and increased catecholamine production at night promotes breakdown of triglycerides into free fatty acids and very low density lipoproteins. However, a consistent association with dyslipidemia and response to treatment with CPAP has not been clearly demonstrated (19).

## Part 3: Disparities in Sleep and Cardiometabolic Health in African Descent Populations

Compared to White Americans, AAs have higher rates of cardiometabolic disease including hypertension, obesity, diabetes, and stroke (62). It is notable that these differences persist after correction for behavioral risk factors, such as diet, physical activity, and smoking (63). It is estimated that 40–50% of the premature mortality experienced by AAs is due to an increased burden of cardiovascular and metabolic disease (64). In view of the physiologic and epidemiologic evidence linking sleep and cardiometabolic disease, it is logical to postulate that disparities in duration and quality of sleep could provide one of the mechanisms contributing to these adverse outcomes.

### Disparities in Sleep Duration in AAs

A number of extended epidemiologic surveys have been conducted, such as the Americans’ Use of Time Survey, examining self-reported sleep at multiple points from 1975 to 2006 (65). AAs consistently reported short sleep more than any racial group over the 31-year interval reported. Ruiters et al. conducted a meta-analysis of 14 studies examining differences in sleep in AAs and Whites and found significant reductions in both objective and self-reported measures of sleep duration in the AA group (66).

Psychosocial factors may have an influence, as total sleep time differences disappeared in Ruiters’ analysis when measured in laboratory versus the home environment, and employment history also moderated the difference. It has been suggested that AAs are more likely to live in an urban environment with adverse influences on sleep from factors, such as crime, crowding, and noise. A cross-sectional evaluation of 5,301 AAs enrolled in the Jackson Heart Study found that lower education was associated with greater odds of long sleep and poorer sleep quality. Higher levels of neighborhood violence and other neighborhood problems were associated with shorter sleep and reduced sleep quality. Adjustment for depression attenuated some but not all of these correlations (67). In a study comparing AA and White residents in the same urban community, sleep duration was not different, suggesting environmental rather than genetic factors influenced sleep duration in this population (1).

While data suggest that social and environmental factors play an important role in creating disparities in sleep patterns in AAs, data are also exploring the contribution of African ancestry to sleep patterns in the Diaspora. In a study of 77 AAs and 101 European Americans who underwent polysomnography, ancestral allele frequencies were calculated to derive the percentage of African admixture proportion (AF%, African allele frequency) (68). A panel of 1,698 ancestry informative markers (i.e., markers that maximally distinguish between ancestral populations) were genotyped. Analysis indicated that AF% was correlated with a lower percentage of slow wave sleep. Sleep duration and sleep efficiency were not associated with ancestry markers.

In a multi-ethnic assessment of genetics determinants of sleep duration, 25,000 individuals of African, Asian, European, and Hispanic ancestry were genotyped for approximately 50,000 SNPs of 2,000 genes. A SNP (rs17601612) in the dopamine D2 receptor gene was significantly associated with sleep duration in European subjects, but with an opposite direction of association in AAs (69). In AA participants in the Jackson Heart Study, genetic analysis found that a SNP in the CLOCK (circadian locomotor output cycles protein kaput) gene, which is important to circadian metabolic pathway regulation, was correlated with short sleep duration and BMI (70).

### Disparities in Sleep Duration Contribution to Cardiometabolic Disease in AA

Studies are now linking the reduced habitual sleep time in AAs to cardiometabolic risk factors (Table 1) (71, 72). Knutson et al. assessed blood pressure and actigraphic sleep duration over a 5-year interval (72). After adjustment for covariates, reduced sleep duration increased odds of incident hypertension and appeared to mediate the difference between AAs and Whites in diastolic blood pressure change over time. Curtis et al. directly examined the correlation between total sleep time and sleep efficiency (per cent time in bed sleeping) with overall cardiometabolic risk in an urban Midwest population. Risk was estimated from a composite of seven biomarkers (blood pressure, waist circumference, hemoglobin A1C, insulin resistance, triglycerides, HDL cholesterol, and C-reactive protein) (63). AAs experienced less sleep and had lower sleep efficiency than expected. After adjusting for sociodemographic and health behaviors, the authors discovered that sleep time and sleep efficiency accounted for 41 and 58%, respectively, of the racial disparity in cardiometabolic risk scores (63).

There are some data that suggest disparities in cardiometabolic effects from short sleep duration may reflect not only a greater prevalence of short sleep but an increased adverse sensitivity to the effects of short sleep. Vishnu et al. reviewed Center for Disease Control national survey data and, while all populations demonstrated an increased risk of coronary heart disease with insufficient sleep, the risk was especially increased in AAs (multivariable adjusted odds ratio for cardiovascular disease in Whites 1.6 versus 2.1 in Blacks, with the confidence interval 1.6–2.6 in the latter) (73). Jean-Louis et al. examined data from the NHIS 1977–2009 (74). After multivariate-adjusted regression, very short (≤5 h) White sleepers had a 10% increased likelihood of being overweight and 51% increased likelihood of being obese. In comparison, very short Black sleepers had a 76% increased likelihood of being overweight and 81% increased likelihood of being obese.

### Disparities in Sleep Disorders in AAs

Conflicting data have been found regarding racial differences in self-reported chronic insomnia, with some studies showing an increased risk in AAs, which is partially attenuated with adjustment for socioeconomic status, but other studies showing a decreased risk (75). Differences in methodology may explain these different results, and Kingsbury et al. has suggested that when analysis is restricted to studies utilizing validated measures with prospective data collection, AAs appear to have a greater prevalence of insomnia (2). However, differences in outcomes could also reflect differences between the cohorts of populations analyzed. Jean-Louis et al. have identified that not only are there differences in sleep complaints between ethnic groups but also potentially differences within an ethnic population (76). In their survey of urban community-residing older adults, Caribbean Americans reported less sleep complaints than US-born AAs, and immigrant European Americans reported greater complaints than US-born European Americans.

Considerable data point to an increase in both prevalence and severity of OSA in AAs relative to Whites. A meta-analysis of 10 studies found a significant increase in prevalence and severity in AAs versus Whites after adjustment for age, gender, and BMI (66). One study found a fourfold to sixfold increase in the prevalence of OSA in AA children relative to White children (77). Greater rates of hypertension and obesity have also been found at the time of diagnosis in AAs, and AAs demonstrate more severe levels of oxygen desaturation during sleep (78, 79). Recently, an examination of inter-individual severity of sleepiness in the presence of sleep apnea found that AAs with OSA demonstrate more severe sleepiness as measured by either the Epworth Sleepiness Scale or Psychomotor Vigilance Testing (80). Severity of sleepiness also corresponded to levels of the proinflammatory cytokine IL-6 in this study. Compounding these findings is evidence that when AAs are referred for evaluation of sleep apnea, the majority either does not complete testing or is not compliant with treatment (81, 82).

Social and environmental factors clearly play important roles in the development of obesity and OSA, as well as access to diagnostic testing and compliance with treatment. However, the genetic contribution to sleep disorders also warrants investigation. Genetic control is thought to influence pathogenic factors in OSA, such as obesity, craniofacial morphology, and ventilatory control patterns (83). Chen et al. have recently conducted genome-wide association studies using cohorts from seven multi-ethnic studies and have identified a locus on chromosome 17 overlapping the RAII gene which associates with non-REM OSA in men but not women (84). These genetic influences likely vary between populations. In the Cleveland Family Study, segregation analysis suggested a co-dominant gene with an allele frequency of 0.14 predicted 35% of the variance of AHI in AAs. Adjustment for BMI strengthened the association, suggesting the genetic influence was independent of genes for obesity. This result was in contrast to European Americans, in whom a recessive inheritance of OSA was suggested and the association was weakened with adjustment for BMI (83).

An analysis of genetic admixture, the San Paolo Epidemiologic Sleep Study, after correcting for sex, age, BMI, and socioeconomic status, found European genetic ancestry was associated with an increased risk of manifesting OSA and, in contrast to United States epidemiologic patterns, African heritage correlated with a surprising lower risk of OSA (85).

### Disparities in Sleep Disorders Contribution to Cardiometabolic Outcomes in AAs

While considerable research is demonstrating the connections between sleep impairment and cardiometabolic disease for the general population, limited data exist specific to African descent populations (Table 2). One study has suggested a possible increased sensitivity to circadian rhythm disruption in AAs versus White Americans. In the Nurses’ Health Study II, rotating night shift work was independently associated with an increased risk of incident hypertension only in AA workers (86). The 2007–2008 National Health and Nutrition Examination Survey found that self-report of either a diagnosis or symptoms of OSA were more strongly associated with hypertension in AAs (OR = 4.7) than in Hispanics (OR = 2.0) or Whites (OR = 1.7) (87).

A number of studies have explored inter-relationships between sleep disturbances and the metabolic syndrome in AAs. Kazman et al. surveyed 248 AAs urban residents and found that symptomatic snoring predicted the presence of the metabolic syndrome as well as fasting glucose and BMI (88). Sleep quality as reflected by the Pittsburg Sleep Quality Index did not. In a cross-sectional analysis of Blacks with metabolic syndrome, 49% were found to be high risk for OSA (89). Sleep apnea risk factors, but not insomnia symptoms or short sleep duration, associated with diabetes and hypertension (90).

In view of the increased prevalence and severity of OSA in AAs, coupled with the challenges in diagnosis and achieving successful treatment, OSA is likely to play an important role in the disparities in cardiovascular outcomes for AAs. Unfortunately, very limited data are available examining the extent to which OSA and other sleep disorders contribute to the increased burden of cardiometabolic disease in AAs and further research is needed. Of particular value in better defining a potential causative role would be interventional studies examining the ability of aggressive screening and treatment for sleep disorders to make an impact on the disproportionate level of cardiometabolic disease experienced by the population. In view of the increased prevalence of sleep apnea in Blacks with metabolic syndrome, Jean-Louis et al. conducted a controlled trial of culturally and linguistically tailored telephone interventions to improve compliance with patients in this population (82). The intervention group was 3.2 times more likely to attend the sleep consultation. Patients in both arms of the study who were diagnosed with sleep apnea demonstrated a high rate of compliance with treatment. Future research should assess the ability of such interventions to make an impact on the disparities in cardiometabolic outcomes for these patients.

## Part 4: Sleep in Africans

### Sleep Patterns in Africans and African Immigrants

Although Africa is the second largest and second most populous continent, limited data are available in regard to sleep patterns and the prevalence of sleep disturbance. A small number of brief surveys have been conducted in selected countries (91, 92). For example, on International Sleep Well Day (March 21, 2002), a standardized questionnaire which included the Athens Insomnia Scale and Epworth Sleepiness Scale was administered in 10 countries worldwide (91). Chosen as the representative of Africa, South Africa was included and rated the second worst country in quality of sleep. Overall, in the 10 countries surveyed, South Africa performed worst in measures of daytime sleepiness, second worst in the level of sleep symptoms, and second highest in category of “not sleeping well” (91).

In 2006–2007, the World Health Organization conducted the INDEPTH WHO-SAGE Study [International Network for the continuous Demographic Evaluation of Populations and Their Health in developing countries (INDEPTH), Study on Global Aging and Adult Health (SAGE)] (92). Face to face interviews were conducted in adults greater than 50 years of age in eight sites in Asia and four sites in Africa: Agincourt (South Africa), Ifakara (Tanzania), Nairobi (Kenya), and Navrongo (Ghana). Sleep problems were assessed by two questions, one regarding frequency of sleep disturbance and the second regarding degree daytime tiredness. Percentage of sleep disturbance ranged from 4% in Kenyan men to 31% of South African women. In all countries, women and older age groups reported greater sleep disturbance. Less education, the lack of a partner, and a poor self-rated quality of life were consistently associated with a higher prevalence of sleep issues.

Studies in Western Europe and America have indicated a prevalence of insomnia in 30–60% of the elderly population. Investigators at the University of Ibadan, Nigeria, conducted a survey of 2,152 residents 65 years of age or older finding a prevalence of 31% (93). Age >70, female gender, an unmarried status, and the presence of depression, chronic pain, or chronic medical conditions were all risk factors.

The question can be asked to what degree variations in sleep patterns in Africa versus North America and Europe might represent the effects of urbanization and industrialization. Yetish et al. conducted an assessment of sleep focused on three preindustrial societies, specifically Tanzania and Namibia in Africa and Bolivia in South America (94). Key findings from all three locations revealed a strong similarity to “modern” humans regarding an average duration of 5.7–7.1 h. There was evidence of varying circadian patterns, as the villagers typically went to sleep several hours after sunset and awoke several hours before sunrise. Additionally, there was substantial seasonal variation resulting in about 1 h less sleep in the summer months than the winter months, as environmental temperature appeared to be an important regulator of sleep duration and timing.

In contrast to this study, when Black African students from the Ivory Coast recorded their sleep patterns in the cool-dry period of January versus the hot-dry period of May, no major seasonal variations were noted other than an increase in awakenings in the warm season (95).

While direct measurements of sleep patterns in Africans are few, there are reasons to predict that sleep disturbance will be an increasingly important public health issue. One factor is the burden of infectious diseases in Africa. HIV/AIDS is a major public health concern and cause of death in many parts of Africa. Sub-Saharan Africa alone accounted for an estimated 69% of all people living with HIV and 70% of all AIDS deaths in 2011 (96). It is estimated that 73% of HIV-positive patients suffer from a poor sleep quality. A study found that 45% of HIV patients sleep less than 6 h per night and 56% of the sample reported fragmented sleeping (97). Cardiovascular disease ranks among the leading causes of death in HIV+ patients in developed countries (98) and one anticipates similar associations to emerge in Africa. It seems likely that the disturbance of sleep in HIV patients contributes to the adverse cardiometabolic outcomes (99).

Africa has the highest incidence of human African trypanosomiasis, also called “sleeping sickness” or Chagas disease (96). Trypanosomiasis infection creates an inflammatory state affecting the central nervous system resulting in detrimental effects on the sleep–wake cycle of these patients and greatly impeded quality of life, referred to by the Zulu term of Nagana (N’gana) to name this disease, which means powerless/useless (96). Malaria is another parasitic disease that is common in tropical countries. While there are currently no human studies that have explored the relationship between sleep and trypanosomiasis or malarial infections, animal studies seem to suggest sleep deprivation is damaging to the immune system and leads to an increased infection severity of malaria parasites (96).

With growing urbanization and changing lifestyles, the incidence of sleep disorders is likely to increase rapidly in Africa. Urbanization without adequate structural and political foundations predisposes to poor and low-income neighborhoods. A major part of the African population is currently living in developing countries with high poverty rates and development indices below the international average. The lack of perceived neighborhood safety due to crime or social instability may contribute to psychological stress and increasing sleep impairment among residents.

Finally, the prevalence of overweight and obesity is increasing among children and adolescents in developing countries, rising from 8.1% in 1980 to 12.9% in 2013 for boys and from 8.4 to 13.4% in girls (100). In some African populations, in adults, the estimated prevalence of obesity exceeds 50% (100). This raises the concern of OSA as a potential emerging health issue.

### Sleep Quality Association With Cardiometabolic Outcomes in Africans

A few studies have explored whether the same sleep–cardiometabolic associations seen in Western countries are also demonstrated in African populations. Cole et al. utilized wrist actigraphy and sleep diaries to assess 263 rural and urban participants in Ghana (101). Objectively measured total sleep time was 7.6 h on average (SD = 1.1) with 38% exhibiting long sleep (more than 8 h per night), 9% sleep longer than 9 h, 29% short sleep (less than 7 h per night), and 10% sleep shorter than 6 h per night. Total sleep time was positively associated with a 10-year cardiovascular disease risk, whereas short sleep, defined as sleeping less than 7 h per night on average, was negatively associated with a 10-year cardiovascular disease risk. However, sleep duration was not associated with prevalent cardiovascular disease or metabolic syndrome.

In one study, a cohort of 329 South African teachers (45% Black) were assessed for risk of OSA, based on questionnaire and neck circumference, and blood prothrombotic factors over a 3-year interval. High risk for sleep apnea correlated with increased thrombotic factors relative to low risk subjects and new onset sleep apnea risk was associated with a rise in thrombotic indicators (102).

A study based in a cardiology clinic in Douala, Cameroon evaluated 411 hypertensive adults for the presence of daytime sleepiness as indicated by the Epworth Sleepiness Scale (103). 63% exhibited daytime sleepiness and this was associated with the presence of diabetes and snoring, suggesting similarities to Western pathophysiologic patterns for this population.

Finally, investigators conducted polysomnography in a group of 20 hypertensive Black South Africans and a closely matched control group without hypertension. Similar to findings in other ethnic populations, the hypertensive group was significantly more likely to exhibit OSA and low arterial oxygen saturations during the night (104).

## Conclusion

Data from many venues and studies strongly support the powerful influence of sleep duration and sleep quality on cardiometabolic outcomes. Evidence that sleep disparities play an important role in the adverse cardiometabolic outcomes experienced by AAs is increasing and warrants further investigation. Major cultural and environmental changes are occurring in Africa and are likely to impact sleep, potentially causing a rising rate of cardiometabolic disease throughout the African continent. Because issues related to sleep duration and sleep quality are potentially addressable, research to explore feasible interventions to improve sleep duration and sleep quality in African descent populations are imperative.

## Author Contributions

PLW, JO, NST and AES conceived of the idea. PLW, JO and NST did the original literature search. PLW, JO, NST and AES contributed to writing the original manuscript. PLW and AES did the critical rewrites.

## Conflict of Interest Statement

The authors declare that the research was conducted in the absence of any commercial or financial relationships that could be construed as a potential conflict of interest.
